# “Pro-dopamine regulation (KB220Z™)” as a long-term therapeutic modality to overcome reduced resting state dopamine tone in opiate/opioid epidemic in America

**DOI:** 10.15761/JSIN.1000129

**Published:** 2016-09

**Authors:** K Blum, F Marcelo, K Dushaj, L Fried, RD Badgaiyan

**Affiliations:** 1 Department of Psychiatry & McKnight Brain Institute, University of Florida College of Medicine, Gainesville, FL, USA; 2 Department of Psychiatry & Behavioral Sciences, Keck School of Medicine of USC, Los Angeles, CA, USA; 3 Division of Applied Clinical Research & Education, Dominion Diagnostics, LLC., North Kingstown, RI, USA; 4 Synaptamine, Inc., Austin, TX, USA; 5 Division of Clinical Neurology, PATH Foundation NY, New York, NY, USA; 6 Division of Personalized Medicine, IGENE, LLC., Austin, TX, USA; 7 Division of Molecular Neurobiology, LaVitaRDS, Salt Lake City, UT, USA; 8 Division of Neuroscience Research and Addiction Therapy, Shores Treatment & Recovery Center, Port Saint Lucie, FL, USA; 9 Department of Clinical Psychology and Addiction, Eötvös Loránd University, Hungary; 10 Department of Psychiatry, Laboratory of Molecular and Functional Imaging, University at Minnesota, Minneapolis, MN, USA

**Keywords:** pro-dopamine regulator, dopamine homeostasis, opiate/opioid epidemic, KB220Z, resting sate dopamine tone

## Abstract

Since it is known that relapse, morality, and hospitalizations have been tied to the presence of the Dopamine D2 Receptor A1 allele, as one example, and carriers of this gene variant have a proclivity to favor amino-acid therapy, it seems intuitive that the incorporation of modalities to provide a balance and or restoration of hypodopaminergia should be considered as a front-line tactic to overcome the current American opiate/opioid epidemic, saving millions from death and unwanted locked-in-addiction. If we continue down the prim road path of fighting addiction to narcotics with narcotics, we are doomed to fail. This lesson can also have global interest.

## Introduction

Addiction to psychoactive drugs poses a significant threat to the health of the social and economic fabric of families, communities, and nations. The number of substance users is staggering. The annual United States National Survey on Drug Use and Health (NSDUH) estimated that in 2013, approximately 24.6 million Americans aged 12 or older used illicit drugs in the past month (Substance Abuse and Mental Health Services Administration [SAMHSA], 2014) [1]. This problem urgently requires the development novel treatments for addiction and advanced methods to evaluate the efficacy of potential therapeutic agents. Developing treatments based on well-known biosynthetic pathways that regulate central dopamine (DA) systems involved in mediating rewarding experiences is a major challenge. To curtail psychoactive drug abuse and dependence, the United States Food and Drug Administration (FDA) approved several pharmaceutical agents collectively known as Medication Assisted Treatment (MAT) [2]. It is noteworthy that these agents have helped a portion of patients over the years; however, they have not fully prevented drug craving and relapse. The limited success of treating psychoactive substance abuse with current modalities leaves open the need to develop new therapies [3–6].

Attention must be focused on the recent changes to the current United States law that now limits physicians who prescribe buprenorphine for the detoxification or management of Opioid Use Disorder, to treating a maximum of 275 patients. However, the present structure does not deliver complete management as defined by the American Society of Addiction Medicine’s (ASAM) criteria. The system also experiences both division and stigma and will need a substantial transformation to observe ASAM’s request for unified delivery of addiction treatments. In a recent article [7]. Blum’s group called for an increase in the patient limit and treatment prescriptions to be limited only to physicians who are Board Certified in Addiction Medicine by the American Board of Addiction Medicine (ABAM) or in Addiction Psychiatry by the American Board of Psychiatry and Neurology (ABPN), or additional responsible clinical institutions. Such organization would include treatment integration, treatment structures, and recovery with the aid of prescription medications. Furthermore, it should supervise emotional blunting, treatment improvements, and the introduction of genetic addiction risk testing.

## After money or after care?

There are an estimated 14,500 institutions and programs in the United States that deliver treatment for all inclusive addictive behaviors under the term, “Reward Deficiency Syndrome (RDS)” [8]. While many of these clinical organizations have respectable intentions of delivering much needed aid to those who suffer from RDS, we offer, herein, that a majority of their efforts, particularly during aftercare, are not founded on existing scientific outcomes [9–12]. We use the term “aftercare” to denote any type of program or therapy after principal treatment, including 12-step programs [13]. In this trieste, we are proposing that a hypodopaminergic trait (genetic) and/or state (epigenetic) is serious in considering prolonged impulse to use/abuse alcohol or other drugs, which can ultimately lead to relapse.

While there is support for FDA-approved drugs to treat drug addiction (e.g., alcohol, opiates, nicotine), these drugs support only short-term assistance via blocking dopamine [14]. We, the authors, contend for the application of long-term assistance, which stimulates “dopamine homeostasis” or in other words, promotes “normalcy.” We propose that this approach could be achieved through several holistic methods including, but not limited to, dopamine-enhancing diets, hyper-oxygenation, toxic heavy metal detoxification, exercise, meditation, yoga, and most notably, balancing brain neurotransmitters utilizing nutraceuticals (i.e., KB220 variants). We certainly embrace 12-step programs and fellowships, but with caution, as they are not a stand-alone method, specifically during aftercare.

It is significant that there is a developing scientific foundation for the explanation of the importance of resting-state functional connectivity (rsFC) and its involvement in RDS treatment [15]. It has been established that drugs, food, smoking, gambling, and even compulsive sexual behavior reduce rsFC [16]. Thus, we must ask the treatment community to consider potential brain reward circuitry restoration to repair this damaged crosslink between different brain areas (e.g., nucleus accumbens (NAc), cingulate gyrus, hippocampus, etc.) to be incorporated into the aftercare plan in all treatment programs in America. Anything less will ultimately lead to the so-called “revolving door” for as many as 90% of treatment participants.

## A molecular neurobiological aspect

Based upon neurochemical and genetic support, we propose that both prevention and treatment approaches of addiction (i.e., alcohol, nicotine and glucose), should encompass a biphasic methodology [17]. Hence, acute treatment should involve preferential blockage of postsynaptic NAc dopamine receptors (D1–D5), while long-term stimulation of the mesolimbic dopaminergic network should trigger and/or release Dopamine (DA) at the NAc site [17]. The inability to do so will affect mood, behavior, and possible suicide ideation. Persons with a lack of serotonergic and/or dopaminergic receptors, and an amplified rate of synaptic DA catabolism due to the catabolic genotype of the COMT gene, are susceptible to self-medicating with substances or behaviors that stimulate DA discharge, such as alcohol, opiates, psychostimulants, nicotine, gambling, sex, and even extreme internet gaming [18]. These substances and/or behaviors provoke feelings of well-being. Unfortunately, sustained and prolonged abuse leads to a toxic “pseudo feeling” of well-being, causing disease or discomfort. Thus, a decrease in DA receptors due to the DRD2 A1 allelic genotype [19] (30–40% less D2 receptors), results in excessive craving behavior; whereas, a normal or sufficient amount of DA receptors results in low craving behavior [20].

In considering the prevention of substance abuse, one potential objective would be to prompt production of DA D2 receptors in genetically pre-disposed persons. While in vivo experiments utilizing a standard D2 receptor agonist produce down-regulation, experiments in vitro have shown that continuous activation of the DA receptor network via a known D2 agonist causes substantial production of D2 receptors, despite genetic antecedents [21]. In essence, D2 receptor stimulation signals negative feedback mechanisms in the mesolimbic system to induce mRNA expression causing proliferation of D2 receptors.

## Proposal: A integrated system approach

The authors suggest that D2 receptor activation can be achieved through the utilization of a biological, but therapeutic, nutraceutical formulation that possibly stimulates DA discharge, affecting the similar stimulation of D2-directed mRNA and thus, production of D2 receptors [22]. This production of D2 receptors will stimulate the reduction of craving behaviors [23]. Indeed, as previously mentioned, this method has been established in the literature, showing DNA-directed compensatory overexpression (a type of gene therapy) of the DRD2 receptors, causing a substantial decrease in alcohol cravings in alcohol-seeking rodents [24] and cocaine self-administration [25]. Using natural dopaminergic repletion therapy to encourage long-term dopaminergic stimulation will lead to a common, safe, and efficient method to treat all types of Reward Deficiency Syndrome (RDS) behaviors (i.e., Substance Use Disorders [SUD], Attention-Deficit/Hyperactivity Disorder [ADHD], obesity, and other reward deficient aberrant behaviors) [26]. This theory is additionally supported by the more inclusive comprehension of the function of dopamine in the NAc as a “wanting” courier in the meso-limbic DA network [27].

## Understanding dopaminergic tone in RDS

It is uncertain whether or not attention-deficit/hyperactivity disorder (ADHD) is a hypodopaminergic or hyperdopaminergic disorder. Various sets of data intimate ADHD as either a hyperactive or hypoactive dopamine network. While indirect approaches utilized in previous research have reached contradictory conclusions, Badgaiyan et al. [28] directly measured the tonic and phasic discharge of dopamine in ADHD subjects. The tonic release in ADHD and control subjects was measured and compared utilizing a dynamic molecular imaging technique. The phasic discharge during the performance of Eriksen’s Flanker Task was measured in the two sets using a single scan dynamic molecular imaging technique. In these experiments, subjects were placed in a positron emission tomography (PET) camera and dispensed a dopamine receptor ligand [11] C-raclopride intravenously. Following the injection, PET data were obtained dynamically, while subjects either remained still (tonic release experiments) or completed the Flanker Task (phasic release experiments). PET data were evaluated to measure dynamic variations in ligand binding potential (BP) and other receptor kinetic factors. The evaluation discovered that at rest, the ligand BP was considerably increased in the right caudate of ADHD subjects, signifying decreased tonic release. During task performance, considerably lower ligand BP was detected in the same region, representing increased phasic release. In ADHD, and potentially all RDS behaviors, tonic release of dopamine is decreased and the phasic release is augmented in the right caudate. By depicting the nature of dysregulated dopamine neurotransmission in ADHD, the outcomes elucidate previous findings of decreased or increased dopaminergic functioning, indicating hypodopaminergia in RDS.

## Neurochemical mechanisms and clinical relevance of pro-dopamine regulation

We are suggesting that the utilization of a Pro-Dopamine Regulator, like KB220Z, naturally mirrors the established Brain Reward Cascade (BRC) [29] whereby, serotonin in the hypothalamus stimulates 5HT2a receptors to induce the release of enkephalins in the hypothalamus. Delta and mu receptors inhibit GABA in the Substania Nigra (GABA A receptors), whereas GABA B receptors regulate the Glutaminergic pathway at the Raphe Nuclei site through MNDA receptors projecting to the Ventral Tegmental Area (VTA). The VTA fine-tunes the correct quantity of dopamine to be released at the NAC into the synapse to stimulate nine receptors, whereby D1 and D2 are the major receptors. This process provides for enhanced well-being (reward and pleasure) and anti-stress (blocking unwanted pain related stress up-regulated molecules such as norepinephrine, corticotropin releasing factor, vasopressin, hypocretin, and substance P) [30–35]. Neuroimaging studies in both humans and rat models have shown that KB220z increases resting state functional connectivity in a default putative network as well as the recruitment of dopamine firing in selective brain reward regions, leading to reduced dopamine deficiency, homeostasis, and reduced opiate/opioid craving [36,37] One other important aspect is that in double-blind human studies, the variant KB220 has been shown to block stress in addicted patients measured via skin–conductance, significantly enhancing well-being, and preventing relapse to psychoactive drugs of abuse including heroin [38,39].

## Conclusion

Since it is known that relapse, morality, and hospitalizations have been tied to the presence of the Dopamine D2 Receptor A1 allele, [40] and carriers of this gene variant have a proclivity to favor amino-acid therapy, [29] it seems intuitive that the incorporation of modalities to provide a balance and/or restoration of hypodopaminergia should be considered as a front-line tactic to overcome the current American opiate/opioid epidemic, potentially saving millions from death and unwanted locked-in-addiction. If we continue down the prim road path of fighting addiction to narcotics with narcotics, and not consider the importance of reward deficiency and anti-reward, we are doomed to fail [41].
